# Effects of Tamsulosin Combined With Solifenacin on Lower Urinary Tract Symptoms: Evidence From a Systematic Review, Meta-Analysis, and Trial Sequential Analysis of Randomized Controlled Trials

**DOI:** 10.3389/fphar.2020.00763

**Published:** 2020-05-26

**Authors:** Yuxuan Song, Guangyuan Chen, Peng Huang, Cong Hu, Xiaoqiang Liu

**Affiliations:** ^1^ Department of Urology, Tianjin Medical University General Hospital, Tianjin, China; ^2^ The Second Clinical Medical School, Nanchang University, Nanchang, China; ^3^ Center for Evidence-Based Medicine, School of Public Health, Nanchang University, Nanchang, China; ^4^ Jiangxi Province Key Laboratory of Preventive Medicine, School of Public Health, Nanchang University, Nanchang, China

**Keywords:** lower urinary tract symptoms, benign prostatic hyperplasia, tamsulosin, solifenacin, systematic review, meta-analysis

## Abstract

This study is aimed to systematically evaluate the efficacy of tamsulosin combined with solifenacin and provide clinical evidence for treatment of benign prostatic hyperplasia (BPH) with lower urinary tract symptoms (LUTS). PubMed, Cochrane Library, EMBASE, China National Knowledge Infrastructure, Chinese Biomedical Literature Database, and Wanfang data information service platform were searched to select randomized controlled trials (RCTs) of tamsulosin combined with solifenacin in the treatment of BPH with LUTS. After extraction of the data, the statistical information was calculated by means of STATA 12.0. The publication bias was calculated using Egger's test and Begg's funnel plot. A total of 17 articles contained 1,870 patients treated with tamsulosin in combination with solifenacin and 1,897 patients treated with tamsulosin only were included in this study. Results show that tamsulosin combined with solifenacin therapy was more effective in reducing the Total International Prostate Symptom Score (TIPSS), Storage International Prostate Symptom Score (SIPSS), Quality of life (QOL), and Overactive bladder symptom score (OABSS) in comparison with tamsulosin monotherapy treatment. However, it was found that the combination therapy may increase levels of prostate-specific antigen (PSA) and the maximal urinary flow rate (QMAX). Differences between the combination therapy and tamsulosin monotherapy were not statistically significant for urgency episodes per 24 h, micturitions per 24 h, Voiding International Prostate Symptom Score (VIPSS), and postvoid residual volume (PVR). Tamsulosin combined with solifenacin therapy is more effective than tamsulosin monotherapy for the treatment of BPH concurrent with LUTS and won't increase the risk of dysuria.

## Introduction

Benign prostatic hyperplasia (BPH) is the most common cause of dysuria in middle-aged and elderly men (Porst et al., 2017). According to recent epidemiological reports, there is a very high occurrence of BPH among men over 50 years old in China with more than 50% of BPH patients having associated lower urinary tract symptoms (LUTS) (Lee et al., 2014). LUTS is usually associated with bladder dysuria including frequent urination, urgency, urinary dysfunction, poor urination, and poor urine flow. Several studies have shown that 8 to 31% of 50-year-old men and 27 to 44% of 70-year-old men have moderate to severe LUTS (Sagnier et al., 1994; Bosch et al., 1995; Fang et al., 2018). A survey showed that 29% of men aged 70 years have had two episodes of nocturnal enuresis, 11% have had more than three episodes of nocturnal enuresis, and 19% of men show varying degrees of urge incontinence (Fowler and Barry, 1993; Hunter et al., 1995). LUTS is recognized as adversely affecting the quality of life (QOL) as well as having other serious consequences such as urinary retention due to urinary tract obstruction (Lytton et al., 1968). LUTS affects not only the male patients but also the spouse and might pose difficulties to the family's daily life and family relationships.

For patients with LUTS complicated by BPH, the current treatment is mainly drug-based, including alpha-1 adrenergic receptor blockers (α1-blockers), cholinergic receptor antagonists, 5α-reductase inhibitors, phosphodiesterase type 5 inhibitors, and β3-receptor agonists (McVary et al., 2011).

Tamsulosin is a highly selective, long-acting α1-blocker that is widely used for treating common diseases of the male genitourinary system. The α1 blockers act mainly on the urethra, bladder neck, and prostate and have a selective blocking effect on smooth muscle in these organs (Sun et al., 2019). Tamsulosin can, therefore, improve LUTS and prevent and treat the urinary retention. In addition, tamsulosin can also be used for urinary calculi and adjuvant treatment of male sexual dysfunction.

However, it has been suggested that a single drug is not the ideal therapy for relieving LUTS, especially as measured by the Storage International Prostate Symptom Score (SIPSS) (Xiao et al., 2012). Solifenacin, a muscarinic acetylcholine M3 receptor blocker (M3-blocker), is an anticholinergic drug with high selectivity. It has been suggested that M3 receptors on the bladder detrusor muscle might be the target of this drug (Chen et al., 2010). Through inhibiting and blocking the binding of acetylcholine to the M3 receptor, reducing the contractile force of the detrusor, and inhibiting contraction of the detrusor, solifenacin can improve the symptoms of frequent urination and urgency (Henningsohn et al., 2017). However, due to the wide distribution of M3 receptors in the body, adverse reactions such as dry mouth, blurred vision, and constipation may occur. In addition, solifenacin may increase the risk of acute urinary retention, a feature of anticholinergic drugs that may limit their clinical usage (Russo et al., 2014).

To address these issues, a combination therapy of M3-blockers and α1-blockers is proposed for the treatment of LUTS/BPH. Recently, there have been a number of published studies exploring the effects of combined tamsulosin/solifenacin therapy on LUTS/BPH. These studies lack consistency and are controversial, with some studies showing that tamsulosin combined with solifenacin therapy versus tamsulosin alone in the treatment of LUTS/BPH is more effective in reducing the Total International Prostate Symptom Score (TIPSS) (Song et al., 2016), QOL (Xianhe et al., 2012), and postvoid residual volume (PVR) (Yuan et al., 2017), while Xiang et al. considered tamsulosin combined with solifenacin therapy *versus* tamsulosin alone in the treatment of LUTS/BPH can only influence the PVR (Xianhe et al., 2012). In addition, others consider there was no statistically significant difference between the two treatments in improving TIPSS (Lee et al., 2017) and QOL (Seo et al., 2011). Most of these studies are based on limited samples, presenting difficulties in drawing rigorous conclusions. In this regard, the present study was conducted to verify whether combined tamsulosin/solifenacin therapy is better than tamsulosin monotherapy in treating LUTS/BPH through systematic review and meta-analysis, as well as providing cases of evidence-based medical guidelines for clinical practice.

## Materials and Methods

### Inclusion Criteria and Exclusion Criteria

The inclusion criteria and exclusion criteria of this systematic review and meta-analysis were summarized in **Table 1**.

**Table 1:** Criteria for considering studies for the review based on the Population, Intervention, Comparator, Outcomes, and Study Designs (PICOS) Structure.

	Inclusion criteria	Exclusion criteria
**Population**	Men satisfying the following criteria were selected for the study: aged more than 40 years; Total International Prostate Symptom Score (TIPSS) of 8 or higher; and BPH diagnosed by ultrasound (Gratzke et al., 2015).	Studies reported on LUTS associated with other diseases but not BPH or neurogenic LUTS; the patients had a history of prostatic surgery or prostate cancer.
**Intervention**	Tamsulosin in combination with Solifenacin therapy.	Other therapy.
**Comparator**	Tamsulosin monotherapy.	Other therapy.
**Outcomes**	TIPSS, SIPSS, VIPSS, QMAX, OABSS, QOL, PSA, Micturitions per 24 h, Urgency episodes per 24 h, and PVR.	Qualitative outcomes such as patient feelings.
**Study Designs**	Randomized Controlled Trials.	Letters, comments, reviews, and other non-randomized studies.

All enrolled studies should follow the criteria: (1) Population: Men satisfying the following criteria were selected for the study: aged more than 40 years; Total International Prostate Symptom Score (TIPSS) of 8 or higher; and BPH diagnosed by ultrasound (Gratzke et al., 2015); (2) Intervention: tamsulosin in combination with solifenacin therapy; (3) Comparator: tamsulosin monotherapy; (4) Outcomes: TIPSS, SIPSS, VIPSS, QMAX, OABSS, QOL, PSA, number of micturitions per 24 h, number of urgency episodes per 24 h, and PVR; and (5) Study Designs: Randomized Controlled Trials (RCTs).

The main exclusion criteria were: (1) Reviews, Meta-analyse, Letters, Case reports; (2) Duplicated studies; (3) Studies not published in either the Chinese or English-language literature; and (4) Reports with incomplete or unavailable data.

### Literature Search

We searched PubMed, Cochrane Library, EMBASE, China National Knowledge Infrastructure, Chinese BioMedical Literature Database, and the Wanfang data information service platform to find relevant studies. The English-language search strategy was (tamsulosin) AND (solifenacin) AND (benign prostatic hyperplasia or BPH) AND (lower urinary tract symptoms or LUTS). We searched studies published before November 1st, 2019. In addition, we also consulted the reference lists of relevant studies to find more relevant studies (**Supplementary Tables 1–4**).

### Outcomes and Measures

Outcomes included: Total International Prostate Symptom Score (TIPSS), Storage International Prostate Symptom Score (SIPSS), Voiding International Prostate Symptom Score (VIPSS), Maximal urinary flow rate (QMAX), Overactive bladder symptom score (OABSS), Quality of life (QOL), Postvoid residual volume (PVR), Urgency episodes per 24 h, Micturitions per 24 h, and Prostate-specific antigen (PSA).

Outcome measures: The symptoms of LUTS/BPH were observed *via* maximal urinary flow rate (Qmax) assessed by uroflowmetry, PVR assessed by ultrasound, and International Prostate Symptom Score (IPSS). There are seven questions in IPSS, and the total score is 35 points. According to the score, LUTS can be divided into three levels. Among them, ≤7 is classified as mild, 8–19 is moderate, and ≥20 is severe (Robert et al., 2018). In the IPSS table, items 2, 4, and 7 are used as SIPSS, items 1, 3, 5, 6 are used as VIPSS (Robert et al., 2018). The amount of urine discharged per unit time is defined as the urinary flow rate, and Qmax is the most significant of the urinary flow rate parameters (Robert et al., 2018). Under normal circumstances, when the urine volume is more than 150 ml, the Qmax of an adult male should be greater than or equal to 15 ml/s, if the Qmax is less than 10 ml/s, it represents obstruction. QOL questionnaire is widely used to measure symptom severity in men with LUTS/BPH. The QOL scoring is 0–6 points, the higher the score, the more severe the patient's symptoms. The highest score is 6 points, in which case the patient feels very distressed about the current symptoms (McKown et al., 2010). OABSS was used to evaluate the degree of storage symptoms. The questionnaire consisted of seven questions, each scored on a five-point scale of 0–4. Prostate volume was assessed by transrectal ultrasound of the prostate, and PSA level was also obtained.

### Statement and Data Extraction

Two evaluators (GC and YS) independently read the retrieved literature and screened out investigations that were not consistent with the study as determined by the title and abstract. When the literature appeared to meet the inclusion criteria, the full text was read carefully to determine whether it would be included in the study. Where opinions differed on documents, a third evaluator (PH) was asked to resolve the dispute.

The following details were extracted from each study by the two authors using a standardized form: name of the first author, date of the publication, area, study design, ethnicity, patient numbers, the number of participants in the experimental group (tamsulosin combined with solifenacin therapy) and control group (tamsulosin monotherapy therapy), dose, and follow-up period.

### Literature Quality Evaluation

The quality of the included RCTs was evaluated by the Cochrane risk bias assessment tool (Ma et al., 2020). The evaluation items include: (a) Methods of random packet sequence generation; (b) Implementation of allocation concealment; (c) Use of blinding; (d) Completeness of the final data; (e) The presence of selective reporting bias; and (f) Presence of additional factors that might contribute to the risk of high bias in the study. Each item was recorded as “high risk,” “unclear,” and “low risk” according to the quality evaluation.

### Statement and Data Extraction

Meta-analysis was performed using international STATA 12.0 software and we chose weighted mean difference (WMD) and 95% confidence interval (95% CI) to calculate pooled results. The Q-test and heterogeneity coefficient *I^2^* were used to assess the heterogeneity between the studies. If there was no obvious heterogeneity (*I^2^* < 50%), we chose fixed effects model (Mantel-Haenszel method) (Mantel and Haenszel, 1959) to combine effect size. If the heterogeneity between studies was very large (*I^2^* > 50%), the random effects model (DerSimonian and Laird method) (Dersimonian and Laird, 1986) was used. Egger's test and Begg's funnel plot were used to evaluate publication bias.

### Sensitivity and Subgroup Analysis

Sensitivity analysis was performed by deleting studies one by one. The combined results showed no significant changes, denoting that the results of this study were relatively steady.

For subgroup analysis, we divided all subject data into different subgroups so that comparisons could be made between subgroups. Subgroup analysis can be performed on different subjects (for example, performed at different times). In the present study, we performed subgroup analysis by evaluating different doses and follow-up periods.

### Meta-Regression

Meta-regression, an extension of subgroup analysis, can analyze the effects of continuous and categorical features, and in principle can analyze the effects of multiple factors at the same time. To explore the causes of heterogeneity, we performed meta-regression to evaluate the influences of multiple factors.

### Trial Sequential Analysis (TSA)

The TSA was performed using the TSA v0.9.5.10 Beta software developed by the Copenhagen Clinical Trial Center in Denmark. This study sets the odds ratio reduction to 20%, the probability of a Type I error at α = 0.05, and the probability of a Type II error at β = 0.2 to calculate the required information size (RIS) (Haase et al.; Hemmingsen et al., 2011). When the population increase is less than expected, the trial sequential monitoring boundary (TSMB) is given based on RIS. We performed this analysis according to the RIS. When the cumulative Z-value crosses the RIS, the results are considered statistically significant. At the same time, it can be considered that the sample size of the accumulated evidence is sufficient.

## Results

### Results of Study Characteristics

**Figure 1:**
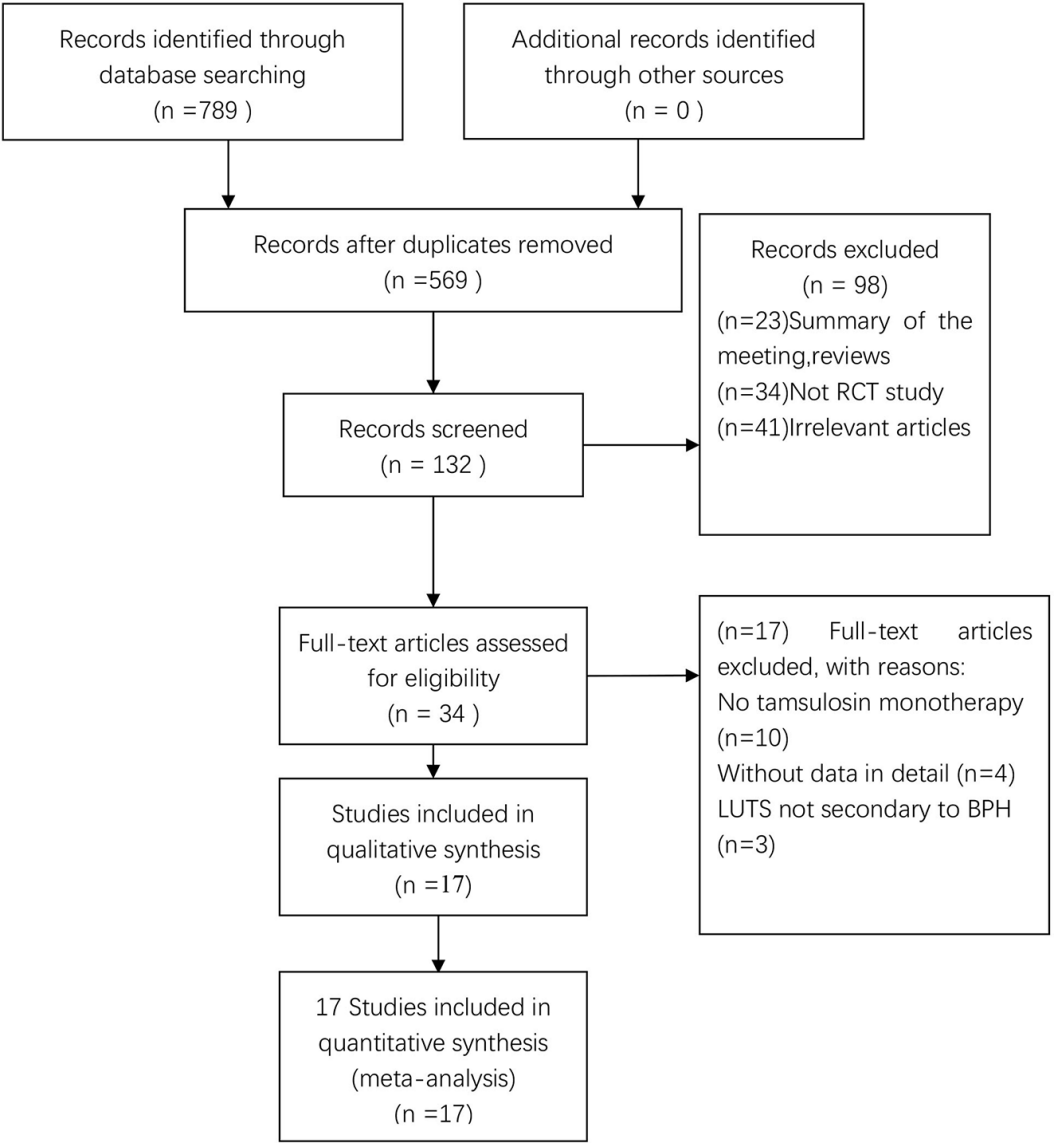
Flowchart illustrating the search strategy.

**Figure 2:**
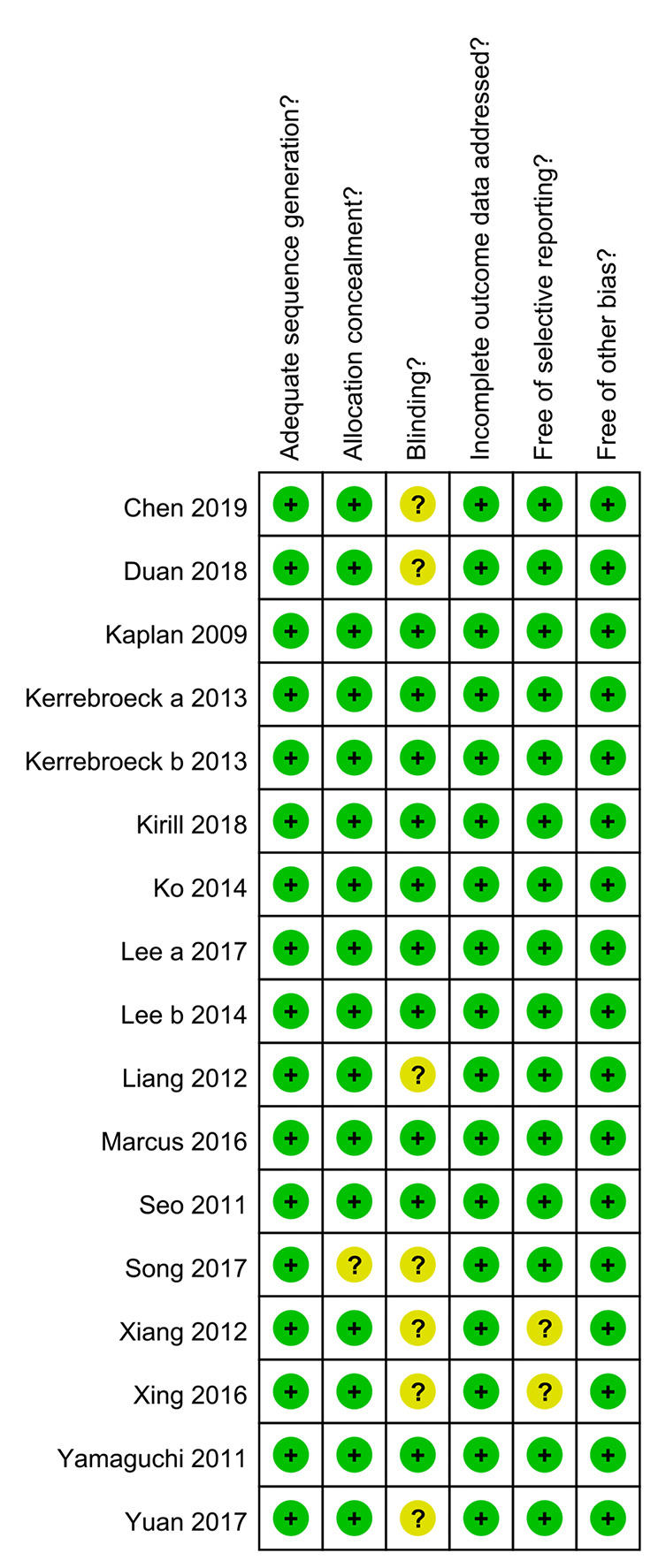
The risk of bias summary.

In total, 789 potentially relevant articles were selected according to the search strategy, which is illustrated in **Figure 1**. Seventeen publications (Seo et al., 2011; Yamaguchi et al., 2011; Xianhe et al., 2012; Yan et al., 2012; Kaplan et al., 2013; Kerrebroeck et al., 2013; Van Kerrebroeck et al., 2013; Lee et al., 2014; Ko et al., 2014; Drake et al., 2016; Song et al., 2016; Yongshi et al., 2016; Hao-ran et al., 2017; Kosilov et al., 2017; Lee et al., 2017; Yuan et al., 2017; Chen et al., 2018) with 3,767 subjects were finally included in our meta-analysis. Among these recruited subjects, 1,870 received tamsulosin combined with solifenacin therapy and 1,897 received tamsulosin monotherapy. Of these studies, 12 were conducted on Asian populations, and 5 on Caucasian populations. The characteristics of the recruited studies are shown in **Table 2**. Among included articles, Lee et al. studied the effects of 5 and 10 mg solifenacin in different groups. Kerrebroeck et al. investigated the effects of 3, 6, and 9 mg solifenacin in different groups. Kirill et al. studied the effects of 5 and 10 mg solifenacin in different groups. We conducted subgroup analysis on the different studies. The risk of bias summary is shown in **Figure 2**. By following the Cochrane Risk of Bias Tool, we found all the 17 RCTs showed the detailed method of random sequence generation and carefully addressed the incomplete data, which indicated they were at low bias in random sequence generation and completeness of the final data. Most studies were free of allocation concealment and selective reporting, while only one study didn't report the detailed method of allocation concealment and two studies didn't report selective reporting. Seven studies didn't state the blinding method. In addition, all the RCTs reported no other bias existed.

**Table 2:** Main characters of recruited studies.

Author	Reference	Country	Ethnicity	Sample size		Dose		Follow-up period
				Combination treatment	Tamsulosin monotherapy	Combination treatment	Tamsulosin monotherapy	
Kerrebroeck ^b^ Philip (2013)	(Kerrebroeck et al., 2013)	Europe	Caucasian	339	327	T (0.4 mg) plus S (6 or 9 mg)	T (0.4 mg)	12 weeks
Yuan (2017)	(Yuan et al., 2017)	China	Asian	32	32	T (0.2 mg) plus S (5 mg)	T (0.2 mg)	4 weeks
Xiang (2012)	(Xianhe et al., 2012)	China	Asian	20	20	T (0.2 mg) plus S (5 mg)	T (0.2 mg)	8 weeks
Liang (2012)	(Yan et al., 2012)	China	Asian	55	53	T (0.2 mg) plus S (5 mg)	T (0.2 mg)	4 weeks
Song (2017)	(Song et al., 2016)	China	Asian	51	76	T (0.2 mg) plus S (5 mg)	T (0.2 mg)	2 weeks
Xing (2016)	(Yongshi et al., 2016)	China	Asian	48	41	T (0.2 mg) plus S (5 mg)	T (0.2 mg)	12 weeks
Duan (2018)	(Hao-ran et al., 2017)	China	Asian	34	34	T (0.2 mg) plus S (5 mg)	T (0.2 mg)	12 weeks
Seo (2011)	(Seo et al., 2011)	Korea	Asian	30	30	T (0.2 mg) plus S (5 mg)	T (0.2 mg)	3 months
Lee ^a^ Kyu (2017)	(Lee et al., 2017)	Korea	Asian	44	55	T (0.2 mg) plus S (5 or 10 mg)	T (0.2 mg)	12 weeks
Yamaguchi (2011)	(Yamaguchi et al., 2011)	Japan	Asian	210	215	T (0.2 mg) plus S (2.5 or 5 mg)	T (0.2 mg) plus placebo	12 weeks
Lee ^b^ Seung (2014)	(Lee et al., 2014)	Korea	Asian	76	80	T (0.2 mg) plus S (5 mg)	T (0.2 mg)	12 weeks
Kaplan (2009)	(Kaplan et al., 2013)	USA	Caucasian	202	195	T (0.4 mg) plus S (5 mg)	T (0.4 mg) plus placebo	4 weeks
Kerrebroeck ^a^ Van (2013)	(Van Kerrebroeck et al., 2013)	Europe	Caucasian	180	179	T (0.4 mg) plus S (3 or 6 or 9 mg)	T (0.4 mg)	12 weeks
Ko (2014)	(Ko et al., 2014)	Korea	Asian	94	93	T (0.2 mg) plus S (5 mg)	T (0.2 mg)	12 weeks
Marcus (2016)	(Drake et al., 2016)	UK	Caucasian	339	327	T (0.4 mg) plus S (6 mg)	T (0.4 mg)	12 weeks
Chen (2019)	(Chen et al., 2018)	China	Asian	53	53	T (0.2 mg) plus S (5 mg)	T (0.2 mg)	12 weeks
Kirill (2018)	(Kosilov et al., 2017)	Russian	Caucasian	93	87	T (0.2 mg) plus S (10 or 5 mg)	T (0.4 mg) plus placebo	10 months

T, Tamsulosin; S, solifenacin. Superscript "a" and "b" were used to distinguish the studies with the similar first author names.

### Meta-Analysis Results of Outcomes

#### Overall Results

##### TIPSS, SIPSS, and VIPSS

**Figure 3:**
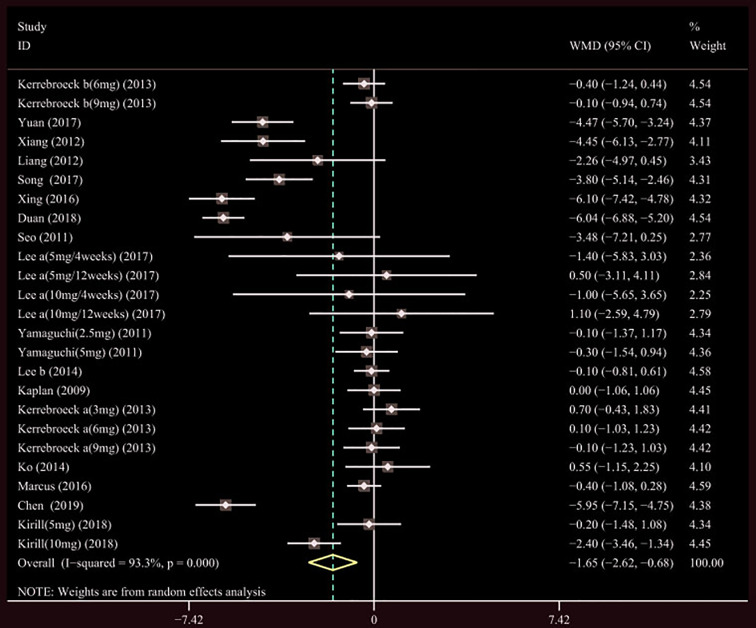
Forest plot of Total International Prostate Symptom Score.

**Figure 4:**
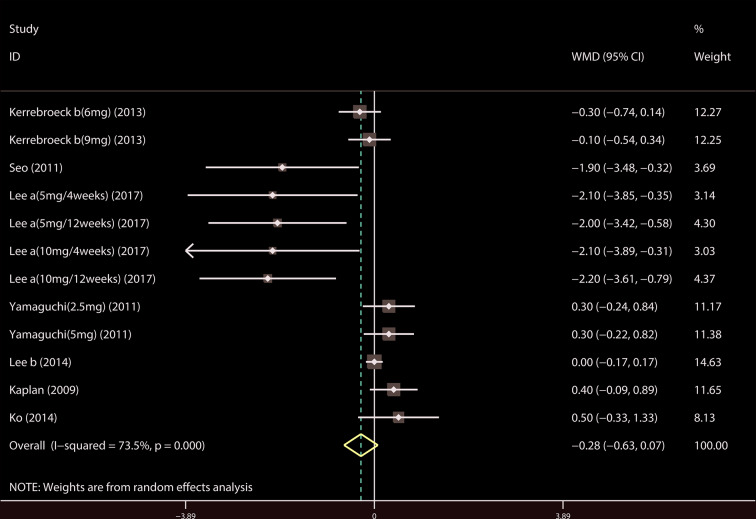
Forest plot of Storage International Prostate Symptom Score.

**Figure 5:**
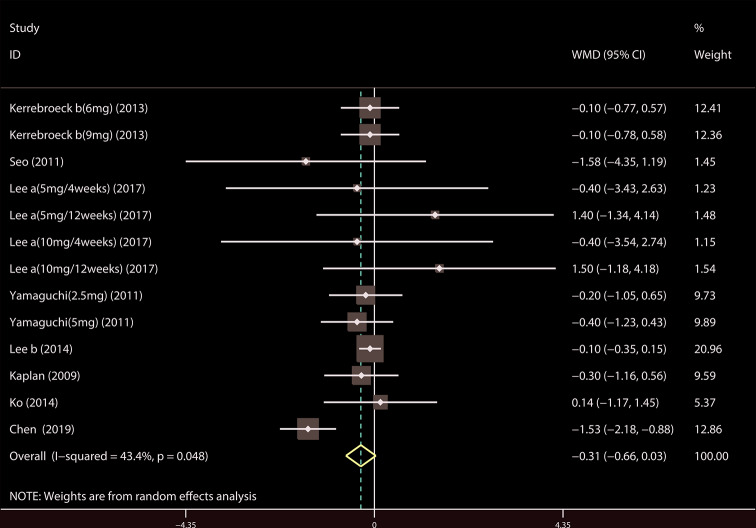
Forest plot of Voiding International Prostate Symptom Score.

A total of 17 (Seo et al., 2011; Yamaguchi et al., 2011; Xianhe et al., 2012; Yan et al., 2012; Kaplan et al., 2013; Kerrebroeck et al., 2013; Van Kerrebroeck et al., 2013; Ko et al., 2014; Lee et al., 2014; Drake et al., 2016; Song et al., 2016; Yongshi et al., 2016; Hao-ran et al., 2017; Kosilov et al., 2017; Lee et al., 2017; Yuan et al., 2017; Chen et al., 2018) studies compared TIPSS. The results showed that the difference between the two groups was statistically significant (WMD = −1.650, 95% CI: −2.617 to −0.682, P = 0.001, **Figure 3** and **Table 3**). Our results indicate that tamsulosin/solifenacin therapy was associated with a greater reduction in TIPSS scores compared with tamsulosin monotherapy. A total of 7 (Seo et al., 2011; Yamaguchi et al., 2011; Kaplan et al., 2013; Kerrebroeck et al., 2013; Ko et al., 2014; Lee et al., 2014; Lee et al., 2017) studies compared SIPSS, and the results showed that the difference between the two groups was not statistically significant (WMD = −0.276, 95% CI: −0.625 to 0.073, P = 0.121, **Figure 4**). A total of 8 (Seo et al., 2011; Yamaguchi et al., 2011; Kaplan et al., 2013; Kerrebroeck et al., 2013; Ko et al., 2014; Lee et al., 2014; Lee et al., 2017; Chen et al., 2018) studies compared VIPSS; the observation indicators are continuity variables while the effect indicators are used by WMD. The results showed that the difference between the two groups was not statistically significant (WMD = −0.311, 95% CI: −0.655 to 0.033, P = 0.076, **Figure 5**).

**Table 3:** Overall meta-analysis results.

Outcomes	WMD (95% Cl)	*P*	Test for heterogeneity		Analysis model	Sample size		Number of study
			*I^2^* (%)	*P*		Combination treatment	Tamsulosin monotherapy	
TIPSS	**−1.650 (−2.617, −0.682)**	**0.001**	93.30%	0.000	R	1,870	1,897	17
SIPSS	−0.276 (−0.625, 0.073)	0.121	73.50%	0.000	R	998	995	7
VIPSS	−0.311 (−0.655, 0.033)	0.076	43.40%	0.048	F	1,051	1,048	8
QMAX	**1.270 (0.266, 2.274)**	**0.013**	93.20%	0.000	R	1,052	981	13
OABSS	**−1.202 (−2.044, −0.361)**	**0.005**	95.60%	0.000	R	613	647	7
QOL	**−0.382 (−0.746, −0.018)**	**0.039**	94.50%	0.000	R	903	905	9
PVR	1.032 (−3.612, 5.676)	0.663	79.70%	0.000	R	1,057	959	9
Urgency episodes per 24 h	0.013 (−0.168, 0.194)	0.888	42.00%	0.069	F	1,186	1,083	6
Micturitions per 24 h	0.145 (−0.156, 0.445)	0.345	56.30%	0.019	R	1,006	996	5
PSA	**0.192 (0.132, 0.253)**	**< 0.001**	0.00%	0.596	F	667	689	6

WMD, Weighted mean difference; CI, confidence interval; R, random effects model; F, fixed effects model; TIPSS, Total International Prostate Symptom Score; SIPSS, Storage International Prostate Symptom Score; VIPSS, Voiding International Prostate Symptom Score; QOL, Quality of life; QMAX, Maximal urinary flow rate; PVR, Post void residual volume; PSA, Prostate specific antigen; OABSS, Overactive bladder symptom score. Bold values meant P-value < 0.05.

##### QMAX, OABSS, and QOL

**Figure 6:**
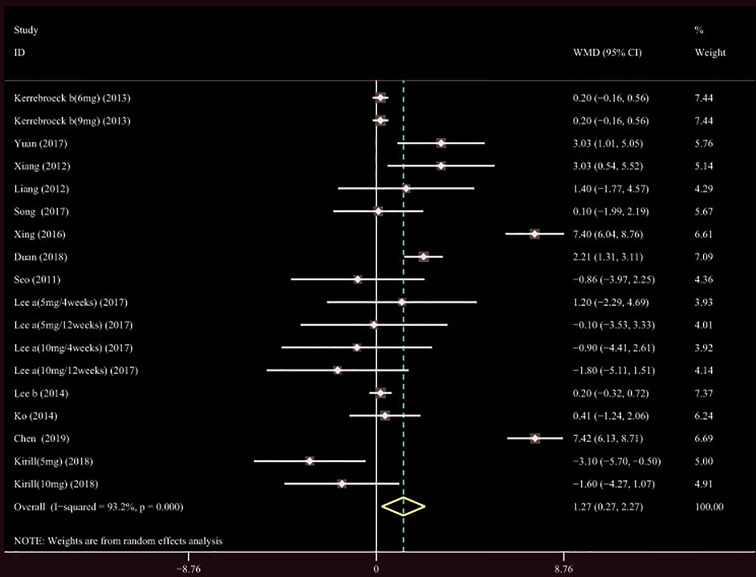
Forest plot of Maximal urinary flow rate.

**Figure 7:**
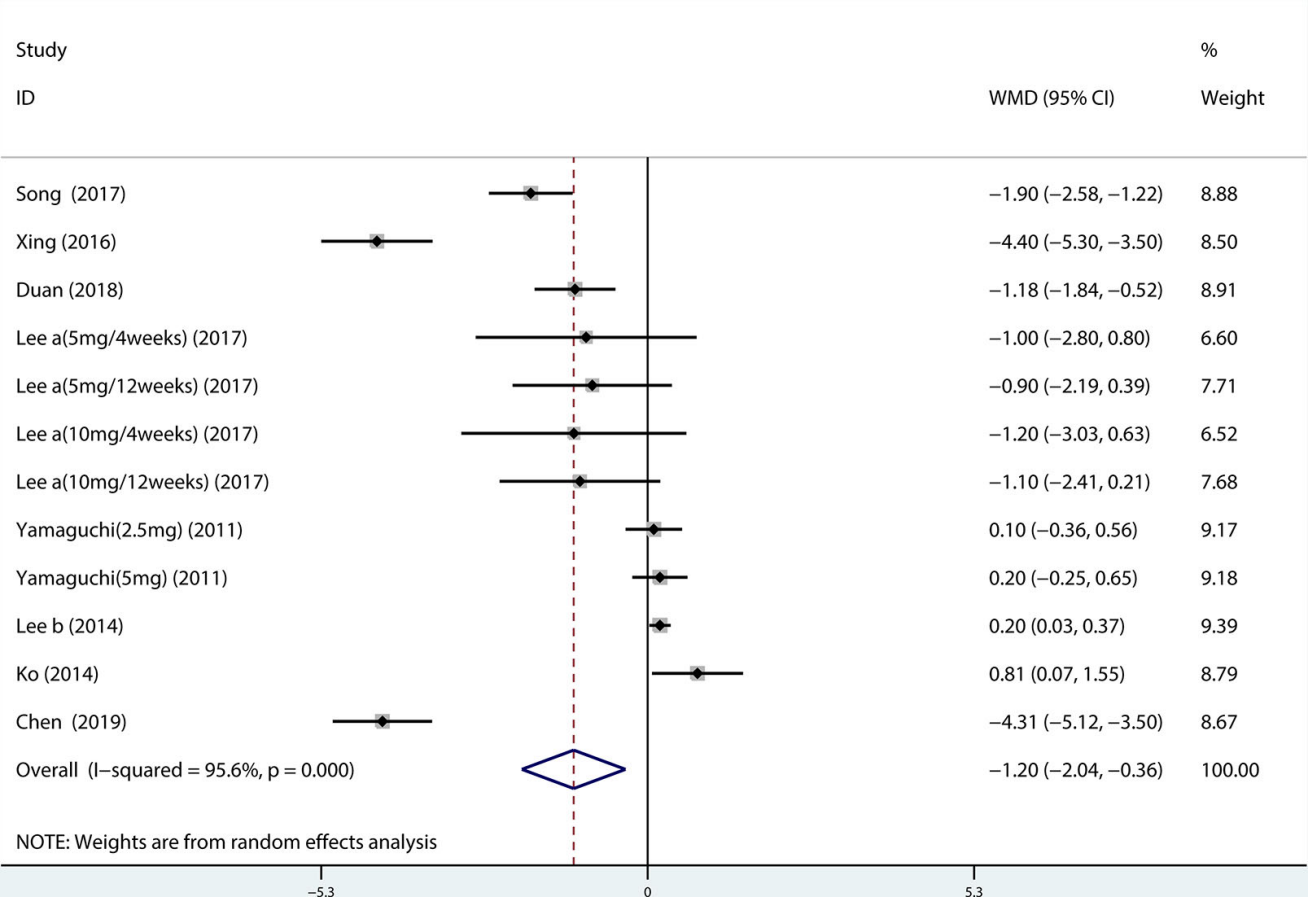
Forest plot of Overactive bladder symptom score.

**Figure 8:**
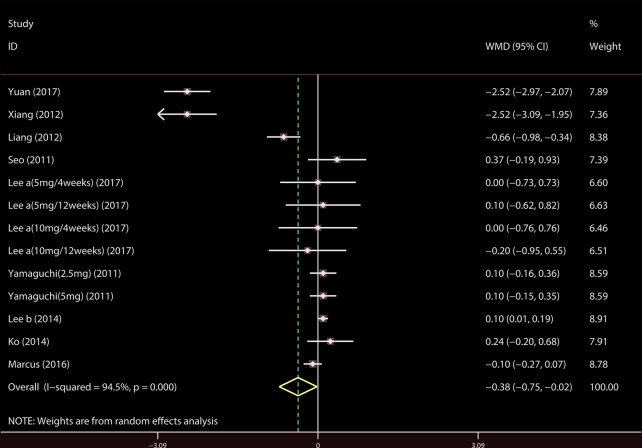
Forest plot of Quality of life.

A total of 13 (Seo et al., 2011; Xianhe et al., 2012; Yan et al., 2012; Kerrebroeck et al., 2013; Lee et al., 2014; Ko et al., 2014; Song et al., 2016; Yongshi et al., 2016; Hao-ran et al., 2017; Kosilov et al., 2017; Lee et al., 2017; Yuan et al., 2017; Chen et al., 2018) studies compared QMAX. The results showed that the difference between the two groups was statistically significant (WMD = 1.270, 95% CI: 0.266 to 2.274, P = 0.013, **Figure 6**). Our results indicated that tamsulosin/solifenacin therapy can increase patients' QMAX compared with tamsulosin monotherapy. A total of 8 (Yamaguchi et al., 2011; Lee et al., 2014; Ko et al., 2014; Song et al., 2016; Yongshi et al., 2016; Hao-ran et al., 2017; Lee et al., 2017; Chen et al., 2018) studies compared OABSS, and the results showed that the difference between the two groups was statistically significant (WMD = −1.202, 95% CI: −2.044 to −0.361, P = 0.005, **Figure 7**). Our results showed that tamsulosin/solifenacin therapy can decrease the OABSS of patients compared with tamsulosin monotherapy. A total of 9 (Seo et al., 2011; Yamaguchi et al., 2011; Xianhe et al., 2012; Yan et al., 2012; Ko et al., 2014; Lee et al., 2014; Drake et al., 2016; Lee et al., 2017; Yuan et al., 2017) studies compared QOL. The results showed that there was a significant difference between the two groups (WMD = −0.382, 95% CI: −0.746 to −0.018, P = 0.039, **Figure 8**). In addition, our results indicated that tamsulosin/solifenacin therapy was associated with a greater reduction in scores for QOL compared with tamsulosin monotherapy. This indicates that, in improving the QOL of patients, tamsulosin and solifenacin combined therapy is considerably more effective than tamsulosin monotherapy.

##### PVR, Urgency Episodes Per 24 h, Micturitions Per 24 h, and PSA

**Figure 9:**
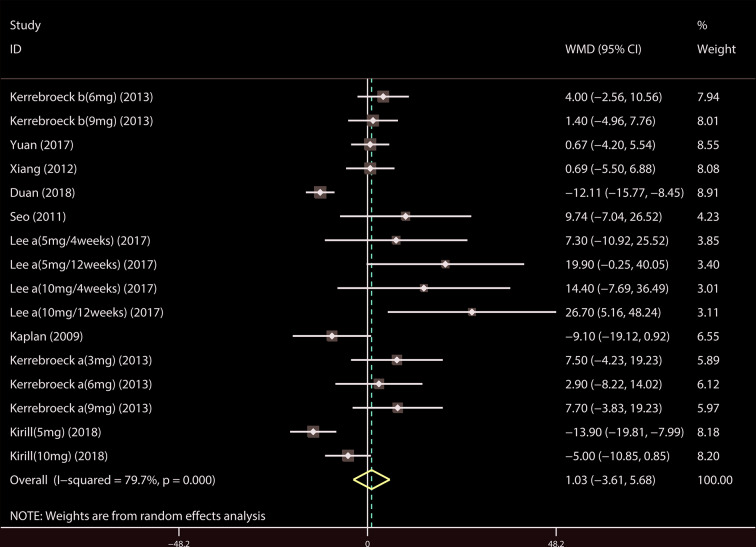
Forest plot of Postvoid residual volume.

**Figure 10:**
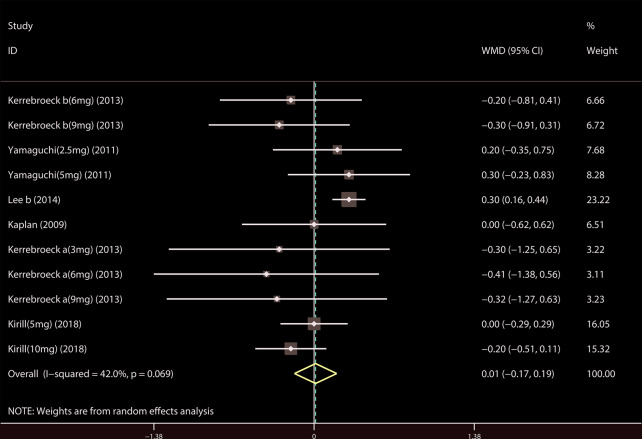
Forest plot of Urgency episodes per 24 h.

**Figure 11:**
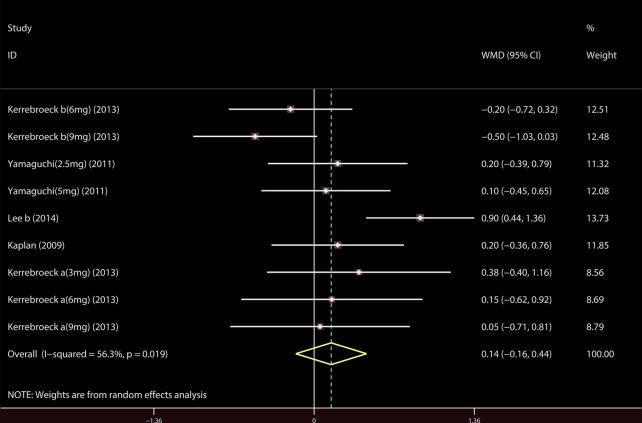
Forest plot of Micturitions per 24 h.

**Figure 12:**
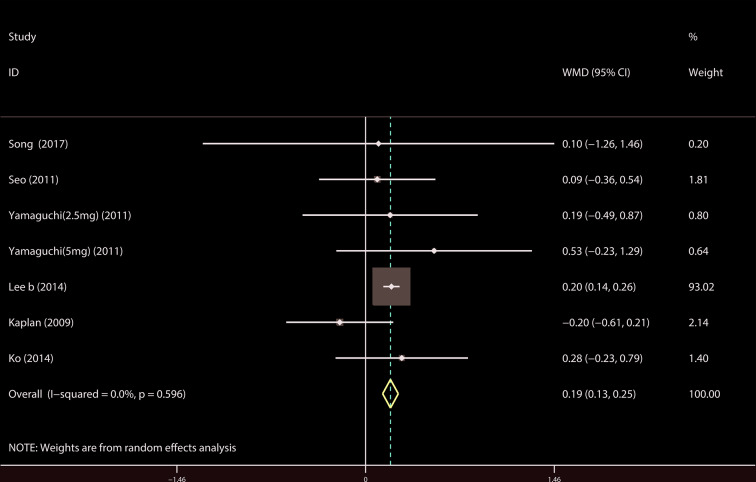
Forest plot of Prostate specific antigen.

A total of 9 (Seo et al., 2011; Xianhe et al., 2012; Kaplan et al., 2013; Kerrebroeck et al., 2013; Van Kerrebroeck et al., 2013; Hao-ran et al., 2017; Kosilov et al., 2017; Lee et al., 2017; Yuan et al., 2017) studies reported the PVR. The results showed that there was no significant difference between the two groups (WMD = 1.032, 95% CI: −3.612 to 5.676, P = 0.663, **Figure 9**). A total of 6 (Yamaguchi et al., 2011; Kaplan et al., 2013; Kerrebroeck et al., 2013; Van Kerrebroeck et al., 2013; Lee et al., 2014; Kosilov et al., 2017) studies were compared with urgency episodes per for 24 h; the observation index was a continuous variable and the effect index was used by WMD. The results showed that there was no significant difference between the two groups (WMD = 0.013, 95% CI: −0.168 to 0.194, P = 0.888, **Figure 10**). A total of 5 (Yamaguchi et al., 2011; Kaplan et al., 2013; Kerrebroeck et al., 2013; Van Kerrebroeck et al., 2013; Lee et al., 2014) studies were compared with the number of micturitions per 24 h; the observation index was continuous variable, and the effect index was used by WMD. The results showed that there was no significant difference between the two groups (WMD = 0.145, 95% CI: −0.156 to 0.445, P = 0.345, **Figure 11**). A total of 6 (Seo et al., 2011; Yamaguchi et al., 2011; Kaplan et al., 2013; Ko et al., 2014; Lee et al., 2014; Song et al., 2016) studies compared the PSA. We found that tamsulosin/solifenacin therapy might increase the level of PSA in patients with PSA compared with tamsulosin monotherapy, which may increase the risk of prostate cancer (WMD = 0.192, 95% CI: 0.132 to 0.253, P < 0.001, **Figure 12**).

#### Subgroup Analysis: Follow-Up Period

In this subgroup analysis, we found that patients in the follow-up period of ≤3 months showed decreased TIPSS, OABSS, and QOL scores. The shorter follow-up time period also had effects on the PSA and number of urgency episodes per 24 h. Patients in the >3 months follow-up period group, however, showed increased QMAX and decreased PVR scores (**Table 4**).

**Table 4:** Subgroup meta-analysis results of follow-up period.

Outcomes	Subgroups	WMD (95% Cl)	*P*	Test for heterogeneity		Analysis model	Sample size		Number of study
				*I^2^* (%)	*P*		Combination treatment	Tamsulosin monotherapy	
TIPSS	≤3 months	−**1.680 (**−**2.729,** −**0.630)**	**0.002**	93.80%	0.000	R	1,777	1,810	15
	>3 months	−1.330 (−3.485, 0.825)	0.227	85.10%	0.009	R	93	87	2
SIPSS	≤3 months	−0.276 (−0.625, 0.073)	0.121	73.50%	0.000	R	998	995	7
VIPSS	≤3 months	−0.311 (−0.655, 0.033)	0.076	43.40%	0.048	F	1,051	1,048	8
QMAX	≤3 months	**1.799 (0.719, 2.878)**	**0.001**	94.20%	0.000	R	959	894	11
	>3 months	**1.973 (3.571, 0.376)**	**0.015**	0.00%	0.524	F	93	87	2
OABSS	≤3 months	−**1.202 (**−**2.044,** −**0.361)**	**0.005**	95.60%	0.000	R	613	647	7
QOL	≤3 months	−**0.382 (**−**0.746,** −**0.018)**	**0.039**	94.50%	0.000	R	903	905	9
PVR	≤3 months	3.138 (−2.011, 8.286)	0.232	78.30%	0.000	R	964	961	7
	>3 months	−**9.439 (**−**18.161,** −**0.717)**	**0.034**	77.30%	0.036	R	93	87	2
Urgency episodes per 24 h	≤3 months	**0.208 (0.086, 0.330)**	**0.001**	23.20%	0.237	F	1,093	996	4
	>3 months	−0.094 (−0.305, 0.116)	0.378	0.00%	0.351	F	93	87	2
Micturitions per 24 h	≤3 months	0.145 (−0.156, 0.445)	0.345	56.30%	0.019	R	1,006	996	5
PSA	≤3 months	**0.192 (0.132, 0.253)**	**<0.001**	0.00%	0.596	F	667	689	6

WMD, Weighted mean difference; CI, confidence interval; R, random effects model; F, fixed effects model; TIPSS, Total International Prostate Symptom Score; SIPSS, Storage International Prostate Symptom Score; VIPSS, Voiding International Prostate Symptom Score; QOL, Quality of life; QMAX, Maximal urinary flow rate; PVR, Post void residual volume; PSA, Prostate specific antigen; OABSS, Overactive bladder symptom score. Bold values meant P-value < 0.05.

#### Subgroup Analysis: Solifenacin Dose

We observed a statistically significant difference between the two groups with dosages of ≤5mg of solifenacin (WMD = −2.209, 95% CI: −3.601 to −0.817, P = 0.002), suggesting that ≤5mg of solifenacin was more effective for decreasing the TIPSS and OABSS (WMD = −1.213, 95% CI: −2.136 to −0.289, P = 0.010) scores. This dose also increased the QMAX, number of micturitions per 24 h, number of urgency episodes per 24 h, and PSA. In addition, we found that 5–10 mg doses of solifenacin are effective for increasing the number of urgency episodes per 24 h (**Table 5**).

**Table 5:** Subgroup meta-analysis results of solifenacin dose.

Outcomes	Subgroups	WMD (95% Cl)	*P*	Test for heterogeneity		Analysis model	Sample size		Number of study
				*I^2^*(%)	*P*		Combination treatment	Tamsulosin monotherapy	
TIPSS	>5 mg	−0.500 (−1.104, 0.103)	0.104	55.10%	0.029	R	875	873	5
	≤5 mg	−**2.209 (**−**3.601,** −**0.817)**	**0.002**	94.50%	0.000	R	995	1,024	12
SIPSS	>5 mg	−0.147 (−0.519, 0.225)	0.439	70.60%	0.002	R	722	710	5
	≤5 mg	−0.800 (−1.757, 0.157)	0.102	81.00%	0.000	R	276	285	2
VIPSS	>5 mg	−0.059 (−0.523, 0.405)	0.581	0.00%	0.712	F	339	327	2
	≤5 mg	−0.417 (−0.898, 0.063)	0.089	58.10%	0.014	R	721	721	6
QMAX	>5 mg	0.166 (−0.087, 0.420)	0.198	0.00%	0.484	F	476	399	3
	≤5 mg	**1.843 (0.097, 3.590)**	**0.039**	94.00%	0.000	R	576	582	10
OABSS	>5 mg	−**1.134 (**−**2.196,** −**0.071)**	**0.036**	0.00%	0.930	F	210	213	2
	≤5 mg	−**1.213 (**−**2.136,** −**0.289)**	**0.010**	96.40%	0.000	R	403	434	5
QOL	>5 mg	−0.100 (−0.266, 0.066)	0.237	0.00%	0.934	F	383	355	2
	≤5 mg	−0.464 (−0.955, 0.027)	0.064	95.90%	0.000	R	520	550	7
PVR	>5 mg	3.437 (−1.904, 8.778)	0.207	54.00%	0.042	R	646	519	4
	≤5 mg	−1.398 (−7.752, 4.957)	0.666	82.80%	0.000	R	411	440	5
Urgency episodes per 24 h	>5 mg	**0.234 (0.001, 0.468)**	**0.049**	0.00%	0.992	F	602	491	3
	≤5 mg	**0.225 (0.107, 0.343)**	**<0.001**	3.00%	0.397	F	584	592	3
Micturitions per 24 h	>5 mg	−0.206 (−0.512, 0.100)	0.187	0.00%	0.483	F	519	447	2
	≤5 mg	**0.409 (0.157, 0.661)**	**0.001**	39.70%	0.156	F	487	549	3
PSA	≤5 mg	**0.192 (0.132, 0.253)**	**<0.001**	0.00%	0.596	F	667	689	6

WMD, Weighted mean difference; CI, confidence interval; R, random effects model; F, fixed effects model; TIPSS, Total International Prostate Symptom Score; SIPSS, Storage International Prostate Symptom Score; VIPSS, Voiding International Prostate Symptom Score; QOL, Quality of life; QMAX, Maximal urinary flow rate; PVR, Post void residual volume; PSA, Prostate specific antigen; OABSS, Overactive bladder symptom score. Bold values meant P-value < 0.05.

### Sensitivity Analysis

**Figure 13:**
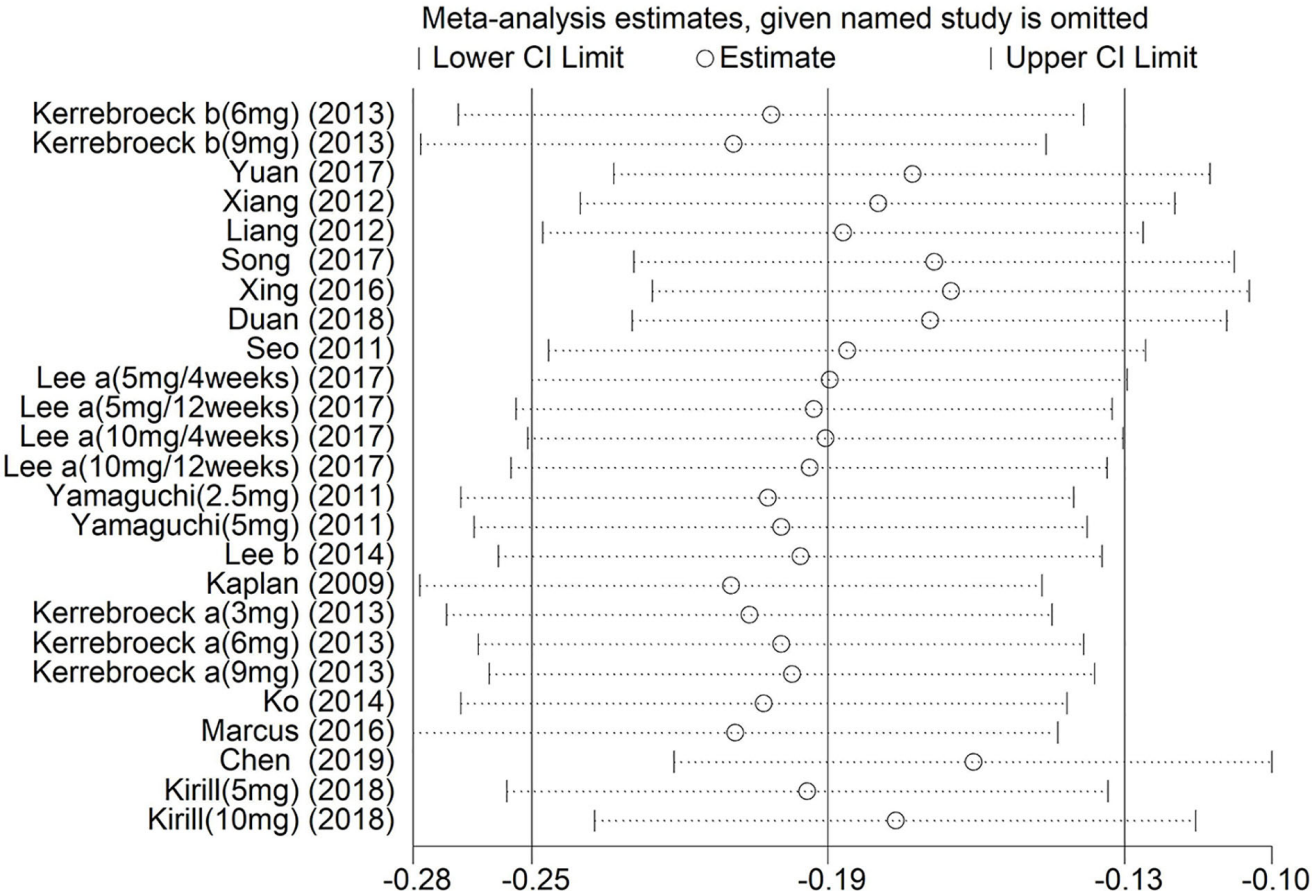
Sensitivity analysis of the pooled ORs and 95% CIs for Total International Prostate Symptom Score.

Sensitivity analysis was implemented by removing one investigation from the meta-analysis at a time. After this process, there was little change in the results (P > 0.05). Moreover, the pooled ORs showed minimal change [−0.25 (Lower Limit) < OR < −0.13 (Upper Limit)], suggesting that our results were reliable (**Figure 13**).

### Publication Bias

Egger's test and Begg's funnel plots were used for publication bias (TIPSS: P = 0.628, VIPSS: P = 0.872, QMAX: P = 0.697, QOL: P = 0.379). Final results indicated that there was no publication bias for the effects of tamsulosin/solifenacin therapy *versus* tamsulosin monotherapy on LUTS in the included studies.

### Trial Sequential Analysis Results

**Figure 14:**
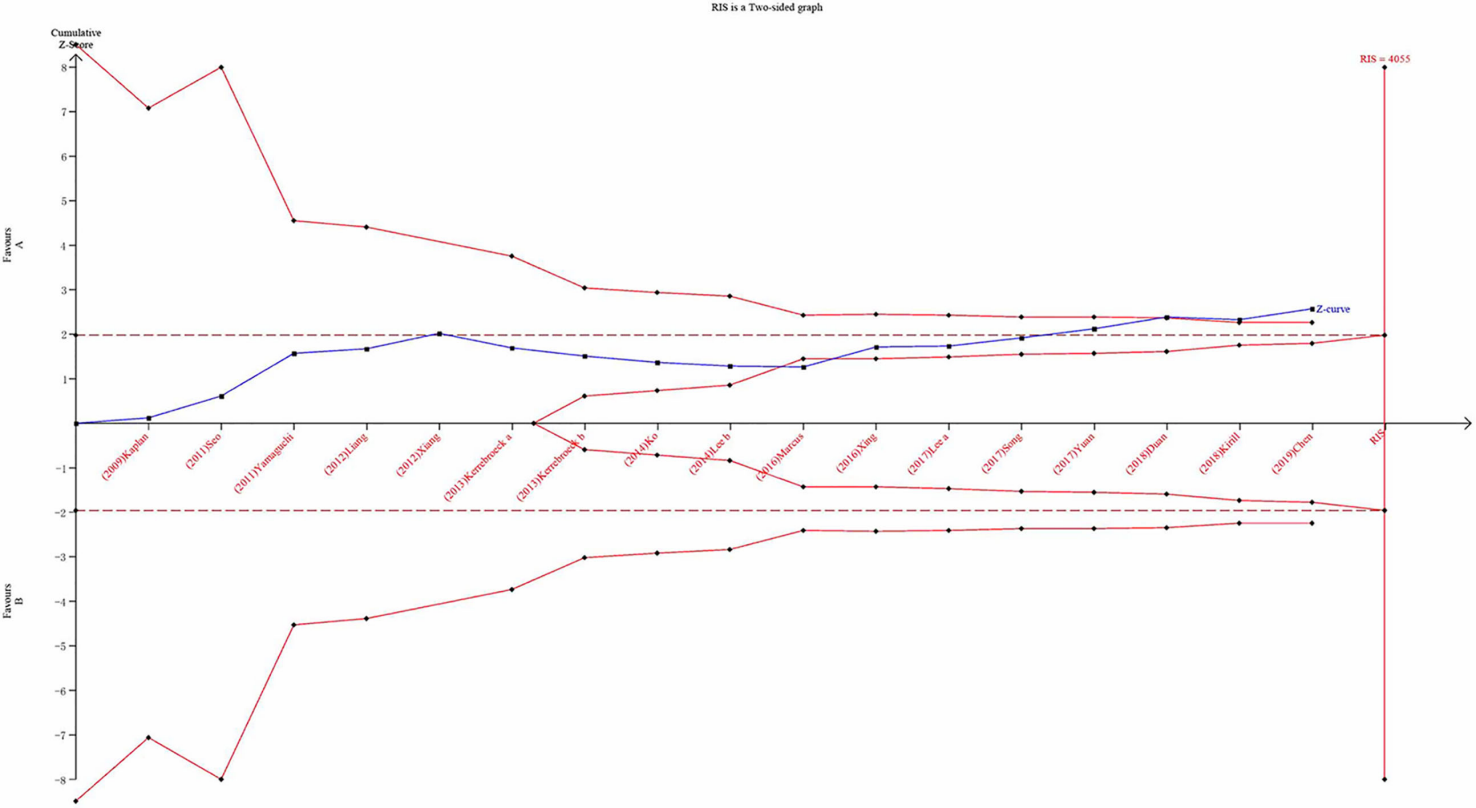
Trial Sequential Analysis results.

We carried out TSA to reduce the risk of type I error and to assess the RIS. Final results indicated that the sample size did not reach the required information size for TIPSS (**Figure 14**; TIPSS: 4,055 cases). A greater number of RCTs are, therefore, required.

### Heterogeneity and Meta-Regression

The heterogeneity is shown in **Table 3**. There is significant heterogeneity in our results. To explore the cause of heterogeneity, we performed meta-regression to evaluate the influences of dose and treatment duration. The results of meta-regression showed that solifenacin dose (P = 0.016) was the source of heterogeneity, while treatment duration (P = 0.093) did not have an impact on heterogeneity.

## Discussion

Our meta-analysis suggested that tamsulosin/solifenacin therapy is more effective than tamsulosin monotherapy for the treatment of LUTS/BPH and does not increase the risk of dysuria. Our results indicated that the combination therapy was associated with decreased scores for TIPSS, QOL, and OABSS compared with tamsulosin monotherapy. In addition, our results showed that the combination therapy was associated with increased scores for QMAX and PSA compared with tamsulosin monotherapy. In the subgroup analysis by dose, we found that ≤5mg solifenacin was more effective for reducing TIPSS and OABSS values as well as for increasing the values of QMAX, micturitions per 24 h, and urgency episodes per 24 h. And that doses of 5–10 mg solifenacin were better at decreasing OABSS. In other words, the dose of ≤5mg solifenacin is effective for treating LUTS/BPH.

In the subgroup analysis according to follow-up period, we found that ≤3 months follow-up period was superior to the >3 months follow-up period in decreasing the TIPSS and OABSS, QOL and had an important effect on the increase in PSA and the number of urgency episodes per 24 h. On the other hand, the >3 months follow-period improved the value of QMAX. In other words, the follow-up period of treatment influences outcome indicators differently. While a short follow-up period (≤3 month) can rapidly improve the TIPSS, OABSS, and QOL, only the longer follow-up period is effective at improving the QMAX.

Our results indirectly suggest that the safety of combining α1-blockers and M3-blockers in the treatment of LUTS/BPH is reliable. The combined therapy did not aggravate urethral obstruction and increased residual urine volume compared with tamsulosin monotherapy. In addition, Van Kerrebroeck et al. (2013), Kerrebroeck et al. (2013) and Drake et al. (2015) found that the incidence of urinary retention was only 1.3% in the tamsulosin/solifenacin therapy group, and 0.6% in the tamsulosin monotherapy group, indicating that there was no significant difference between the two groups. The results in these three studies (Kerrebroeck et al., 2013; Van Kerrebroeck et al., 2013; Drake et al., 2015) are consistent with the results of our meta-analysis.

However, for PSA, the tamsulosin/solifenacin therapy may elevate the PSA levels of patients compared to tamsulosin monotherapy, which may increase the risk of prostate cancer. We suspect that this may account for the stimulating effects of the drugs on the prostate.

In men over the age of 60, LUTS is the most common cause of lower urinary tract obstruction (Gong et al., 2015). LUTS is essentially divided into symptoms of urinary storage, symptoms of urination, and symptoms after urination. Symptoms during storage of urine include frequent urination, urgency, urinary incontinence, and nocturia (Montorsi et al., 2011). The symptoms during urination include anterior urination, thinning of the urinary tract, weakness of urination, and intermittent urination. Symptoms after urination include urinary incontinence and post-urine drip (Caulfield and Birdsall, 1998). Currently, drug treatment with α-blockers and cholinergic receptor antagonists predominate for LUTS patients. The purpose of the medical treatment is to relieve symptoms, relax the smooth muscle of the prostate and bladder neck, relieve urinary tract obstruction, and prevent the occurrence of urinary retention (Filson et al., 2013). Generally speaking, when patients have symptoms of urinary storage, α-blockers should be used. For patients with BPH, timely addition of 5α-reductase inhibitors can significantly increase the effects of these drugs (Caulfield and Birdsall, 1998). This suggests that, for the treatment of LUTS, it is not sufficient to only treat the slack muscle of the prostate. Muscarinic receptors include M1, M2, M3, M4, and M5, among which the M2 and M3 subtypes predominate in the human bladder (Wang et al., 2014). In particular, the M3 receptor directly controls the contraction of the bladder. The M3 receptor plays an important role in regulating bladder smooth muscle and blocking the M3 receptor can reduce the physiological threshold of bladder activity. This indicated that M3-blockers are effective for treatment of LUTS (Gong et al., 2015).

Only one article described the adverse reactions of the drug therapy. This was conducted by Xing et al. (Yongshi et al., 2016), where they found no significant difference in adverse reactions between the experimental and control groups. The overall incidence of adverse reactions in the experimental group during the urine storage period was 10.1% (9/89), of which 2.2% (2/89) was dizziness, 4.5% (4/89) was dry mouth, and 3.4% (3/89) was blurred vision. The overall incidence of adverse reactions in the control group during the urination period was 10.1% (8/79), of which dizziness was 3.8% (3/79), dry mouth was 1.3% (1/79), blurred vision was 2.5% (2/79), and difficulty urinating was 2.5% (2/79). The difference between the incidence of adverse reactions in the experimental and control groups was not statistically significant (P > 0.05). Thus, the combination of the two drugs does not appear to induce more adverse reactions in patients than the tamsulosin monotherapy but can improve the LUTS of patients.

In terms of patient satisfaction, one study (Drake et al., 2015) investigated the satisfaction of patients with tamsulosin in combination with solifenacin. According to the research, more than 80% of patients in the solifenacin 6 mg dose group and in the 9 mg solifenacin dose group were satisfied with the safety of the combination therapy. However, for our meta-analysis results, the dose of 5 or 10 mg solifenacin was found to be effective in treating LUTS/BPH. In terms of efficacy, more than 80% of patients were satisfied the combination therapy. In general, weighing the pros and cons, we believe that the combination therapy for male LUTS patients is effective and can be well tolerated.

Our meta-analysis included 17 studies comprising 1,870 patients with combination treatment and 1,897 patients with tamsulosin monotherapy. A previous analysis by Gong et al. (2015) only had seven articles with 2,167 subjects. These authors found an increase in PVR in the combination therapy compared to the monotherapy group. However, we did not find any significant differences in PVR in the combination therapy compared to the monotherapy group, even in the dose and follow-up period subgroups. In our opinion, the number of studies that Gong et al. included were insufficient, which might be the reason for the difference between their results and ours.

Our meta-analysis also has some advantages. Firstly, our research carried out subgroup analysis by dose, revealing the effects of dose on therapy. Secondly, our research discussed both the safety and satisfaction of tamsulosin and solifenacin combined therapy. Thirdly, compared with a previous meta-analysis, ours contained a larger sample size of 3,767 subjects with 1,870 experimental cases and 1,897 control participants, which is sufficient to draw a reliable conclusion. In addition, sensitivity analysis was performed through removing one investigation from the pooled analysis every time. After this process, we found there was little change in the pooled results (P > 0.05), indicating the pooled results and conclusions were proved to be credible.

There are several unavoidable limitations to this study. Firstly, heterogeneity is very important and might affect the meta-analysis results. In the present meta-analysis, some outcomes had significant heterogeneity. We used random effects model to calculate pooled results and used meta-regression to evaluate the influence of dose and treatment duration. The results of this meta-regression showed that dose (P = 0.016) might be the source of heterogeneity. We therefore carried out the subgroup analysis in order to decrease the heterogeneity and found that the heterogeneity decreased in dose subgroups. Secondly, due to the limited number of articles included, there is no detailed analysis of the safety aspects of the combined drugs. Therefore, more multicentric studies with large sample sizes are still needed in future to confirm our results.

## Conclusions

Overall, the results of this meta-analysis indicated that tamsulosin/solifenacin therapy is superior to tamsulosin monotherapy in the treatment of LUTS/BPH. This combination therapy also does not increase the risk of dysuria. For follow-up period, a short follow-up period (≤3 month) was observed to rapidly decrease the TIPSS, OABSS, and QOL values. For dose, we found that ≤5mg of solifenacin was more effective for decreasing the TIPSS and OABSS and for improving QMAX, the number of micturitions per 24 h, and the number of urgency episodes per 24 h. This indicates that tamsulosin combined with a low dose of solifenacin will benefit patients in the short term. More large-sample, high-quality multicenter RCTs are expected to be carried out in the future.

## Data Availability Statement

The raw data supporting the conclusions of this article will be made available by the authors, without undue reservation, to any qualified researcher.

## Author Contributions

Study design with methodology checking and data analysis guidance: XL and PH. Data collection: GC, YS, and CH. Data analysis: GC and YS. Writing: XL, GC, and YS. Grammar check: YS, GC, and XL.

## Funding

This work was supported by Zhao Yi-Cheng Medical Science Foundation (ZYYFY2018031).

## Conflict of Interest

The authors declare that the research was conducted in the absence of any commercial or financial relationships that could be construed as a potential conflict of interest.
