# Supplementary material for: Effects of Tamsulosin Combined With Solifenacin on Lower Urinary Tract Symptoms: Evidence From a Systematic Review, Meta-Analysis, and Trial Sequential Analysis of Randomized Controlled Trials
Source: Front Pharmacol. 2020 May 26;11:763. doi: 10.3389/fphar.2020.00763 (PMC7264120; doi:10.3389/fphar.2020.00763)
Supplement: Supplementary file 1 [file DataSheet_1.pdf]

(Supplementary Tables 1-4)

Supplementary Table 1 – The detailed search strategy in PubMed
#1 "Tamsulosin" [Title/Abstract]
#2 "Solifenacin Succinate"[Mesh]
#3 "Solifenacin" [Title/Abstract]
#4 #2 OR #3
#5 "Prostatic Hyperplasia"[Mesh]
#6 "Benign Prostatic Hyperplasia"
#7 "BPH"
#8 #5 OR #6 OR #7
#9 "Lower Urinary Tract Symptoms"[Mesh]
#10 "LUTS"
#11 #9 OR #10
#12 #1 AND #4 AND #8 AND #11

Supplementary Table 2 – The detailed search strategy in Cochrane Library
#1 MeSH descriptor: [Lower Urinary Tract Symptoms] explode all trees
#2 LUTS
#3 #1 OR #2
#4 MeSH descriptor: [Prostatic Hyperplasia] explode all trees
#5 Benign Prostatic Hyperplasia
#6 BPH
#7 #4 OR #5 OR #6
#8 MeSH descriptor: [Tamsulosin] explode all trees

#9 MeSH descriptor: [Solifenacin Succinate] explode all trees
#10 Solifenacin
#11 #9 OR #10
#12 #3 AND #7 AND #9 AND #11

Supplementary Table 3 – The detailed search strategy in EMBASE
#1 " tamsulosin ".mp. [mp=title, abstract, full text, keywords, caption text]
#2 " solifenacin ".mp. [mp=title, abstract, full text, keywords, caption text]
#3 " benign prostatic hyperplasia ".mp. [mp=title, abstract, full text, keywords, caption text] OR " BPH ".mp. [mp=title, abstract, full text, keywords, caption text]
#4 " lower urinary tract symptoms ".mp. [mp=title, abstract, full text, keywords, caption text] OR " LUTS ".mp. [mp=title, abstract, full text, keywords, caption text]
#5 #1 AND #2 AND #3 AND #4

Supplementary Table 4 – The detailed search strategy in China National Knowledge Infrastructure, Chinese BioMedical Literature Database and Wanfang data
#1 "坦索罗辛 "[关键词]
#2 " 索利那新 " [关键词]
#3 " 前列腺增生 "[关键词]
#4 " BPH "
#5 #3 OR #4
#6 " 下尿路症状 "[关键词]
#7 " LUTS "
#8 #6 OR #7
#12 #1 AND #2 AND #5 AND #8
